# A Qualitative Study of Cytomegalovirus Awareness and Experience in Kidney Transplant Recipients

**DOI:** 10.1155/joot/1290788

**Published:** 2026-05-26

**Authors:** Afolarin A. Otunla, Ailey McLeod, Katie Gilchrist, Cecilia Vindrola-Padros, Reza Motallebzadeh

**Affiliations:** ^1^ Research Department of Surgical Biotechnology, University College London, London, NW3 2PP, UK, ucl.ac.uk; ^2^ Department of Targeted Intervention, University College London, London, W1W 7TY, UK, ucl.ac.uk; ^3^ Institute of Immunity and Transplantation, University College London, London, NW3 2PP, UK, ucl.ac.uk; ^4^ Department of Nephrology and Transplantation, Royal Free Hospital, London, NW3 2QG, UK, nhs.uk

**Keywords:** cytomegalovirus, experience, kidney transplant, qualitative, treatment

## Abstract

**Objectives:**

To assess the experiences of CMV among kidney transplant recipients in a single transplant centre, focusing on their awareness of CMV, the impact of the disease on their quality of life, and their perceived effectiveness of anti‐CMV therapy.

**Subjects/Patients (or Materials) and Methods:**

We carried out semistructured interviews with 50 adult recipients who had undergone kidney transplantation within the last two years in our centre. Participants were divided into those who had experienced CMV (CMV group, *n* = 25) and those who had not (non‐CMV group, *n* = 25). Data were collected and analysed using inductive qualitative methods, with embedded quantitative descriptive analysis.

**Results:**

Awareness of CMV was low, particularly among those who had not experienced the virus. 32% (*n* = 8) of the non‐CMV group were aware of CMV, compared to 88% (*n* = 22) in the CMV group (*p* < 0.001). Most CMV‐affected participants (78%) reported no symptoms, and the impact on mood and social life was minimal. CMV treatments were rated highly effective, with a preference for pre‐emptive management. There was a strong consensus on the need for better patient education about CMV, with suggestions for more direct communication through healthcare professionals.

**Conclusion:**

This study is the first to provide a comprehensive evaluation of CMV awareness and management from the perspective of kidney transplant recipients in the UK. We highlight a significant gap in CMV awareness among kidney transplant recipients, emphasizing the need for improved educational efforts. Enhanced patient education, particularly through multimedia patient educational tools, could bridge the knowledge gap and ensure better management of CMV in this population. These insights are vital for shaping future patient care strategies and could significantly impact clinical practice, particularly in post‐transplant care.

## 1. Introduction

Cytomegalovirus (CMV) is one of the most common opportunistic infectious complications of solid organ transplantation, with a 1‐year incidence of up to 31.3% in donor positive, recipient negative (D+/R‐) populations and 3.2% in donor negative, recipient negative (D‐/R‐) populations [1]. It may manifest across a clinical spectrum from asymptomatic viraemia to nonspecific symptoms such as fever, night sweats, fatigue, and myalgia and ultimately to tissue‐invasive diseases including retinitis, colitis, and pneumonitis [2]. Post‐transplant infection with CMV is associated not only with the aforementioned sequalae but can also significantly increase the risk of graft rejection, premature graft failure, and mortality [3] and is associated with considerable healthcare resource utilization and costs [4]. To combat this, significant time and resources are spent optimising CMV diagnosis and treatment, either in the form of universal prophylaxis with oral antiviral therapy (for a finite period, usually 3 months post‐transplantation) or pre‐emptive therapy (where surveillance protocols are used to detect early asymptomatic viral replication), as outlined in several national guidelines [5].

Despite its ubiquity and potentially devastating consequences [6–11], there is currently no data exploring the kidney transplant patient understanding and experience of CMV in the UK. Collecting and implementing patient feedback are fundamental in providing patient‐focused care and improving the sense of patient well‐being and are essential in the transplant population due to the importance of their sustained engagement with healthcare services and medication adherence to obtain optimal patient and graft survival [12–14]. By conducting an in‐depth qualitative study interviewing 50 kidney transplant recipients under the care of the renal transplant team at the Royal Free Hospital, London, UK, we aimed to explore patient knowledge and awareness of CMV and its management, draw themes from open‐ended responses about concerns regarding CMV infection, and determine to what extent CMV infection has impacted quality of life.

## 2. Methods

### 2.1. Interview Guide Development

This study was approved by the Royal Free Hospital Institutional Board as a quality improvement project (“Impact on Patient Experience of Cytomegalovirus Infection After Kidney Transplantation”, reference: RFHBU_66322/23).

We designed 2 separate semistructured interview guides based on discussions with patients and renal transplant healthcare physicians and in collaboration with the Rapid Research, Evaluation and Appraisal Laboratory (RREAL) at the Department of Targeted Intervention, UCL. The RREAL team have extensive experience in the integration of qualitative research approaches in clinical studies.

The interviews included closed‐ and open‐ended questions, using a 10‐point Likert scale. We used a semistructured interview guide that followed a narrative and chronological style exploring three predetermined domains: awareness of CMV and its management, effect of CMV infection on quality of life, and perceived efficacy of current treatment. Interviews began with a component assessing quality of life as to contextualize our findings, based on gold standard quality of life assessments such as the Kidney Disease Quality of Life (KDQOL) instrument and short form‐36 health survey questionnaire. The interview guide can be found in the Supporting Information as Supporting Figure 1 (S1).

### 2.2. Participant Selection

Participants were eligible for interviews if they (1) had undergone a renal transplant at the Royal Free Hospital between January 2022 to January 2024, (2) have a currently functioning renal allograft, (3) have not had transplants of any other organ nor an additional kidney transplant prior to survey enrolment, and (4) understood and spoke English without the assistance of an interpreter. Participants were recruited using purposive sampling to ensure variation in the sample. Recruitment began via telephone conversations, where the reasons for the research and a basic introduction regarding the interviewers’ role in the project were discussed. Patient consent was recorded verbally, and information sheets were administered to the participants via email correspondence within 24 h of the initial telephone conversation.

Participants experiencing at least one episode of CMV viraemia and/or clinical CMV infection were allocated to the “CMV group”. Patients who had not experienced a clinically significant CMV viremia at the time of participant selection were allocated to the “non‐CMV group”. Participants were selected to minimise age and gender discrepancies between CMV and non‐CMV groups.

### 2.3. Data Collection

Fifty telephone, virtual, and face‐to‐face (utilizing Microsoft Teams version 24256.2503.3156.9924) semistructured individual interviews were conducted between November 2023 and June 2024. All interviews were audio‐recorded and transcribed using both the Microsoft Teams transcription service (Redmond, Washington, US), and Cockatoo (Tempe, Arizona, US), an AI transcription service. Transcriptions were then cross‐referenced with audio recordings, to ensure verbatim transcription. One individual conducted the interviews (AO) and two researchers conducted the transcription and deidentification. AO is a clinician‐researcher, with experience in transplant medical research. AO had no prior clinical relationship with participants prior to the interviews.

Patient demographics and transplant‐related data were collected through retrospective chart review. We adapted the Consolidated Criteria For Reporting Qualitative Health (COREQ) guidelines to a qualitative survey analysis and we report this alignment in Supporting Figure 2.

### 2.4. Qualitative Analysis

Open coding was carried out to facilitate exploratory analysis. Iterative coding was then performed to create categories and subcategories. Open coding was conducted by one researcher (AM) using an inductive approach. An initial subset of transcripts was coded to generate preliminary codes, which were then reviewed by a second researcher (AO). The coding framework was refined through discussion, with emerging categories and higher‐order themes discussed between AO and AM. An inductive method was chosen because there is no prior research on this topic from which a deductive framework could be developed, and it was important to develop a coding approach based on the actual content of the data itself. After the codes were applied consistently across the dataset, these were grouped into larger themes. Illustrative quotes were selected to represent the main topics included in each theme. Coding and theme development were performed manually, without the use of qualitative data analysis software.

A word cloud was generated as a visual aid to illustrate commonly used terms across interviews. This was not used as an analytic tool and did not inform theme development, which was conducted through inductive qualitative analysis.

### 2.5. Quantitative Analysis

Patient characteristics were described using mean ± standard deviation (SD) if continuous and parametric, median (interquartile range [IQR]) if nonparametric, and count (percentage) if categorical. Baseline characteristics were compared between patients based on CMV status, using the Student’s *t*‐test or Wilcoxon rank sum test, as appropriate, for continuous variables and *χ*‐square test for categorical or discrete variables. Quantitative descriptive statistics, including median (IQR) for nonparametric variables and count (percentage) for categorical variables, were used to perform embedded quantitative descriptive analysis to contextualize and support interpretation of the qualitative findings.

## 3. Results

Of the 167 patients approached, 50 (29.9%) agreed to be interviewed. Reasons for declining participation were as follows: deceased (*n* = 3), did not have time (*n* = 10), not well enough to be interviewed (*n* = 2), did not speak English well enough to understand and respond to the survey questions (*n* = 13), and could not be contacted by telephone after three attempts (*n* = 89). Interviews were conducted via Microsoft Teams in 15 CMV patients and 22 non‐CMV patients and by telephone call in 10 CMV patients and 3 non‐CMV recipients. The interval between transplantation and date of interview was 611 days in non‐CMV recipients and 515 days in patients who developed CMV viraemia (*p* = 0.045). Of note, there was no difference in socioeconomic deprivation, based upon the index of multiple deprivation (IMD), between the two groups. The median length of time of interview in the CMV group was 33.2 min, compared to 22.2 min in the non‐CMV group (*p* < 0.001). Data saturation (the point where no new topics are mentioned by participants) was achieved at *n* = 15 interviews in each group [15]. A comparison of the demographic characteristics of participants who participated in the interviews is shown in Table 1. We began with a component assessing quality of life, before exploring three predefined themes: general awareness of CMV and its management in the context of transplantation, effect of CMV infection on quality of life, and efficacy as well as tolerability of current treatment.

**TABLE 1:** Participant characteristics in recipients with CMV and without CMV infection (non‐CMV).

Variable	CMV (*n* = 25)	Non‐CMV (*n* = 25)	*p* value
*Recipient details*			
Age at interview, years; median (Q1–Q3)	52.6 (45.1–68.5)	58.8 (47.9–70.6)	0.3
Elderly ≥ 65, *n* (%)	9 (36)	11 (44)	0.73
BMI (kg/m^2^); median (Q1–Q3)	24.7 (21.2–28.6)	26.1 (22.7–27.9)	0.85
Male sex, *n* (%)	18 (72)	15 (60)	0.55
Ethnicity, *n* (%)			
White	15 (60)	14 (56)	0.7
Black African	1 (4)	4 (16)	0.7
Black Caribbean	4 (16)	3 (12)	0.7
South Asian	4 (16)	3 (12)	0.7
East Asian	1 (4)	1 (4)	0.7
Obese, BMI ≥ 30 (*n*, %)	4 (16)	3 (12)	1
Dialysis length prior to transplantation, days; median (Q1–Q3)	661 (190–1100)	686 (354–1071.5)	0.55
Current smoker, *n* (%)	3 (12)	1 (4)	0.6
Previous smoker, *n* (%)	11 (44)	9 (36)	0.78
IMD decile; median (Q1–Q3)	5 (3–8)	4 (4–7)	0.88

*Cause of end-stage kidney disease, n (%)*			
Chronic glomerulonephritis	4 (16)	2 (8)	0.13
Hypertension	2 (8)	4 (16)	
Polycystic kidney disease	4 (16)	3 (12)	
Type 1 diabetes	2 (8)	0	
Type 2 diabetes	1 (4)	6 (24)	
IgA nephropathy	1 (4)	3 (12)	
Reflux nephritis	1 (4)	0	
Lupus nephritis	1 (4)	0	
Focal segmental glomerular sclerosis	4 (16)	1 (4)	
Others	5 (20)	6 (24)	

*Transplant details*			
CMV IgG status, *n* (%)			< 0.002
Donor positive/recipient positive	8 (32)	10 (40)	
Donor positive/recipient negative	12 (48)	1 (4)	
Donor negative/recipient negative	1 (4)	7 (28)	
Donor negative/recipient positive	4 (16)	7 (28)	
Age at transplant, years; median (Q1–Q3)	49 (42–64)	56 (46–68)	0.19
Donor age, years; median (Q1–Q3)	48 (38–57)	52 (47–61)	0.4
Donor male sex, *n* (%)	11 (44)	9 (36)	1
Donor ethnicity, *n* (%)			
White	21 (84)	21 (84)	
Black	2 (8)	2 (8)	
Others	2 (8)	2 (8)	
Donor status, *n* (%)			
Live	5 (20)	7 (28)	0.69
DBD	9 (36)	14 (56)	
DCD	11 (44)	4 (16)	
Seen in transplant consent clinic, *n* (%)	12 (48)	12 (48)	1
KDPI; median (Q1–Q3)	0.47 (0.3–0.71)	0.58 (0.35–0.87)	0.64
KDRI; median (Q1–Q3)	0.97 (0.82–1.24)	1.02 (0.84–1.4)	0.74
HLA mismatch level, *n* (%)			0.15
1	0	3 (12)	
2	5 (20)	4 (16)	
3	10 (40)	5 (20)	
4	10 (40)	13 (52)	
Warm ischaemic time, minutes; median (Q1–Q3)	37 (27–44)	35 (28–37)	0.11
Cold ischaemic time, minutes; median (Q1–Q3)	853.5 (652–1099)	690.5 (478–862)	0.11
Length of surgery, minutes; median (Q1–Q3)	191 (212–679)	203 (580–623)	0.73
Biopsy‐proven acute rejection, *n* (%)	3 (12)	2 (8)	1.0

*Interview details*			
Length of interview, minutes; median (Q1–Q3)	33.2 (27.78–40.67)	22.2 (14.62–28.32)	< 0.001
Interview by MS Teams, *n* (%)	15 (60)	22 (88)	0.07

*Note:* HLA, human leucocyte antigen with level of mismatch defined according to UK allocation policy for deceased‐donor kidneys and based on the mismatch between donor and recipient at the HLA‐A, HLA‐B, and HLA‐DR loci: level 1 was a 000 HLA‐A, HLA‐B, and HLA‐ DR mismatch; level 2 was a 0 HLA‐DR plus 0/1 HLA‐B mismatch; level 3 was a 0 HLA‐DR plus 2 HLA‐B mismatch or a 1 HLA‐DR plus 0/1 HLA‐B mismatch; and level 4 was a 2 HLA‐DR or a 1 HLA‐DR plus 2 HLA‐B mismatch [16].

Abbreviations: BMI, body mass index; DBD, donation after brain death; DCD, donation after circulatory death; IMD, index of multiple deprivation; KDPI, kidney donor profile index; KDRI, kidney donor risk index.

### 3.1. Quality of Life

Each interview began with an exploration of participant general health and wellbeing to contextualize participants’ perspectives on CMV. In line with gold standard quality of life assessments KDQOL and SF‐36, this assessment focused on the impact of health on mood, work, and social life (Table 2).

**TABLE 2:** The impact of participant health on quality of life assessed across three domains: work, mood, and social life.

Domain	CMV (*n* = 25)	Non‐CMV (*n* = 25)	*p*‐value
*Impact on work*			
Number of participants employed at the time of interview. *n*, (%)	8 (32)	7 (28)	1
Number of participants retired at the time of interview. *n*, (%)	3 (12)	5 (20)	0.7
Number of participants no longer working at the time of interview. *n*, (%)	14 (56)	13 (52)	1
Number of participants no longer work due to health limitations. *n*, (%)	7 (28)	5 (20)	0.74
Number of participants working with health‐related restrictions. *n*, (%)	5 (20)	5 (20)	1

*Impact on mood*			
Number of participants who described their mood as positive. *n*, (%)	18 (72)	18 (72)	1
Number of participants who described their mood as low. *n*, (%)	6 (24)	5 (20)	0.87
Number of participants who described their mood as extremely low. *n*, (%)	1 (4)	2 (8)	1

*Impact on social life*			
Number of participants who reported that their health did not impact their social life. *n*, (%)	17 (68)	17 (68)	1
Number of participants who reported that their health did impact their social life. *n*, (%)	8 (32)	8 (32)	1
Number of participants who reported that they had to adjust their social life due to their health. *n*, (%)	4 (16)	5 (20)	1
Number of participants who found social reintegration challenging due to their health. *n*, (%)	3 (12)	3 (12)	1

Overall, participants described themselves as being in good health, though this was often interpreted through the lens of their transplant experience, making it difficult to distinguish transplant‐related health from general wellbeing.

“Yeah, my health has been good. I′ve been able to keep an active life, working, going away on holiday, exercise. Yeah, so it′s been good. Felt good in myself for the last few years, which has been good.” (Non‐CMV10, Male, 57 years)

#### 3.1.1. Impact on Work

Of the 50 participants, 15 (30%) were working at the time of interview and 8 (16%) were retired (Table 2). The 27 remaining (54%) had not returned to work, with 12 (24%) stating they were not working due to health limitations. Among those working, 10 patients (66%) had adapted their roles to accommodate their health, by working from home, reducing hours, or avoiding physical strain. A smaller group (*n* = 5, 33%) described no health‐related restrictions on their work. Those unable to work cited fatigue, comorbidities, or the demands of frequent hospital appointments as barriers. There was no statistically significant difference between CMV and non‐CMV participants in terms of return to work (*p* = 1, Table 2).

“(Does your health impact your work?) It can do. It can do. Yeah, I mean, I don′t always feel great, but that′s why I chose to do the Open University anyway, because I knew I wouldn′t be able to handle like going to uni every day so it′s definitely more suitable to me.” (Non‐CMV44, Female, 28 years)

#### 3.1.2. Impact on Mood

Most participants (*n* = 36, 72%) reported stable or positive mood post‐transplant, often linked to improved wellbeing and relief from dialysis. Several described themselves as naturally “optimistic” or “resilient” and viewed the transplant as life‐enhancing. However, 11 participants (22%) described periods of low mood or anxiety, often triggered by illness episodes, medication side effects, or fear of graft loss. A small number (*n* = 3, 6%) described feeling emotionally overwhelmed during acute illness or postoperative recovery. While emotional experiences varied, most participants (*n* = 39, 78%) felt well supported and confident in managing their mental health day to day. There was no statistically significant difference between CMV and non‐CMV participants in terms of mood (*p* = 0.87, Table 2), with 6 participants (24%) in the CMV cohort reporting low mood or anxiety, compared to 5 participants (20%) in the non‐CMV cohort.

“(Does your health impact your mood?) No, mood′s not been affected. I, fortunately was blessed to be very positive, and nearly all the time I am” (CMV17, Male, 72 years)

#### 3.1.3. Impact on Social Life

The majority of the participants (*n* = 34, 68%) reported no or minimal impact of their health on their social life. These individuals described maintaining social contact, attending events, or travelling post‐transplant. However, 16 participants (32%) experienced limitations to social engagement, citing reasons such as fatigue and anxiety about infection risk. There was no difference in the reported impact of health on social life between CMV and non‐CMV cohorts (Table 2), with 8 participants in each cohort describing limitations on their social life. Of these 16 participants, 9 (18%; 4 in the CMV cohort and 5 in the non‐CMV cohort) described adjusting rather than withdrawing—by socializing at home, avoiding crowded venues, or pacing activities. Social reintegration after prolonged periods of dialysis, illness, or COVID‐19 was mentioned by 6 participants (12%; 3 in each cohort) as a challenge. Nonetheless, most felt they could maintain meaningful social connections, even if the format or frequency had changed.

“(does your health impact your social life?) I mean, yes and no. In terms of, you know, I′ve had to make a few adjustments in terms of, uhm, kind of, well, I can eat and kind of scheduling around medication and that kind of stuff. But you know, just generally kind of stay a social and as active as possible.” (CMV4, Male, 67 years)

### 3.2. General Awareness of CMV in Context of Renal Transplantation

#### 3.2.1. Poor Overall Awareness of CMV

Overall awareness of CMV in the context of renal transplantation was poor (Supporting Table 1), with 20 participants (40%) not remembering receiving information about CMV prior to transplantation (Table 3), even though 24 participants (48%) had been seen in a dedicated consent clinic prior to their transplant. There was no statistically significant difference in CMV awareness between participants who attended the consent clinic and those who did not. Among the 24 participants who attended, 8 (33%) did not recall receiving information about CMV prior to transplant, compared with 12 (46%) of the 26 participants who did not attend (*p* = 0.56).

**TABLE 3:** Summary of participant responses to key interview questions on CMV awareness.

Interview questions	CMV (*n* = 25)	Non‐CMV (*n* = 25)	*p*‐value
Number of participants who worried about the impact of CMV on their overall health. *n*, (%)	16 (64)	15 (60)	1
Number of participants who worried about the impact of CMV on their kidney transplant. *n*, (%)	14 (56)	12 (48)	0.79
Number of participants aware of CMV at the time of interview. *n*, (%)	22 (88)	8 (32)	< 0.001
How worried were participants about CMV on a 10‐point Likert scale; median (Q1–Q3)	2.5 (1–4.375)	0 (0–1)	< 0.001
Number of participants who thought they would recognise the symptoms of CMV. *n*, (%)	6 (24)	1 (4)	0.1
Number of patients who thought more should be done to educate transplant recipients about CMV. *n*, (%)	19 (76)	22 (88)	0.7
Number of patients who were aware of the difference between prophylactic and pre‐emptive anti‐CMV management. *n*, (%)	6 (24)	3 (12)	0.46
Number of patients who thought that more should be done to explain the difference between prophylactic and pre‐emptive approaches. *n*, (%)	18 (72)	10 (40)	0.045

As part of this clinic, the potential risks of transplantation are discussed in detail, including a discussion regarding the risk of developing CMV. The median time from consent clinic appointment to kidney transplant was 312 days, with median time from consent clinic to date of interview 838 days. Of note, all recipients on the waiting list for renal transplantation in our centre are given a patient information booklet as well as access to online education resources, detailing the benefits and risks of transplantation.

**FIGURE 1:**
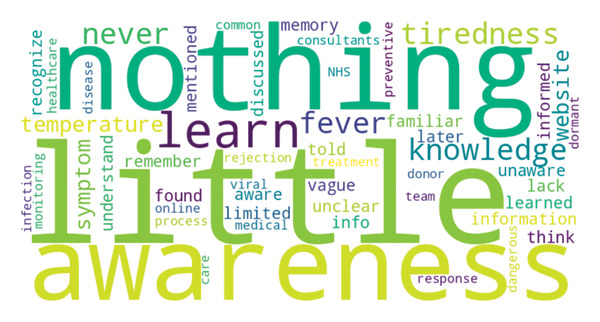
Word cloud of terms used by participants to describe their knowledge and awareness of CMV. The figure was generated by analysing interview transcripts using Python’s “WordCloud” library (version 1.8.1) on October 29^th^ 2024. The size of each word is proportional to its frequency, ranging from *n* = 1 (“vague”) to *n* = 18 (“nothing”).

Figure 1 presents the most common words used to describe participant knowledge of CMV during their interview. Patient awareness of CMV was largely contingent on experiencing infection. In the CMV group, a much higher proportion of participants (*n* = 22/25; 88%) reported that they had heard of CMV at the time of interview, compared to participants who did not develop CMV viraemia (*n* = 8/25; 32%; *p* < 0.001). However, only 20% (*n* = 5/25) of the CMV cohort were aware of CMV prior to developing viraemia.

“I only knew about CMV is when they did a blood test and that′s when they said that I′ve got CMV and obviously got it from the transplant. So yeah, I didn′t know about it before.” (CMV3, Male, 41 years)

#### 3.2.2. Limited Knowledge of CMV Symptoms and Potential Adverse Sequelae

Only one participant (2%) reported that they understood CMV was a virus capable of establishing lifelong latency within an infected individual, whilst 6 participants (12%) were aware of its increased risk profile in the immunosuppressed, but none were aware of the specific risks that it could pose to the transplanted kidney or on overall mortality.

“Well I know that it′s a generally harmless virus which a lot of people carry but for people who are immunosuppressed it can be dangerous and that′s why it′s being taken seriously in my case.” (CMV24, Male, 58 years)

Few patients were aware of the symptoms of CMV, with 7/50 (14%) reporting that they would recognize the symptoms in themselves. Several participants cited this lack of knowledge as a cause for concern. However, some were less worried about not being able to specifically recognize a CMV infection, as they would reach out to their transplant team if they had any health concerns. The source of this knowledge was typically from medical professionals (26/50; 52%) or websites (8/50; 16%).

“(Are you worried that you don’t recognise the symptoms) “Yes because I might have it and then I won′t know but until I have a serious symptom going on and then I end up in hospital which I don′t want to happen”” (CMV5, Male, 72 years)

#### 3.2.3. Concerns About CMV

Participants rated their overall worry as 2.5/10 on our 10‐point Likert Scale, citing potential risk to the health of their kidney transplant as the main cause of concern (14/50, 28%). Whilst there was no statistically significant difference in the proportion of patients who were concerned about CMV in the CMV vs non‐CMV cohorts (64% vs 60%, *p* = 1), there was a clear difference in the level of concern. In the CMV cohort, the median concern about CMV was 2.5 out of 10 (with 0 meaning no worry at all and 10 reflecting extreme worry) compared to 0.0 out of 10 in the non‐CMV cohort (*p* < 0.001).

#### 3.2.4. Suggestions for Patient Education About CMV

82% of participants (41/50) agreed that more should be done to inform transplant patients about CMV, though 8% (4/50) suggested it should be up to the individual patients’ discretion as they may not want to be overwhelmed with more information.

“(Should more be done to educate patients about CMV)Yeah of course as I didn′t know anything about this.” (Non‐CMV9)

One‐on‐one conversations with healthcare professionals (34/50, 68%) were the most popular suggestion for methods through which patients could be informed about CMV. Several patients suggested a combined approach of both a conversation and printed material about CMV.

“I think a conversation and a leaflet because, you know, a lot of the NHS websites are hard to navigate. So, you know, maybe elderly people, maybe people with disability issues, whatever, it′s going to be hard. So a verbal conversation is always going to be the best thing. And then follow up with some information that they can maybe take with them.” (CMV12, Male, 48 years)

Regarding timing, 40% (*n* = 18) of participants thought it would be best to inform about CMV after the transplant, compared to 15.6% (*n* = 7) before the transplant and 20% (*n* = 9) who thought education should be held both pre‐ and post‐transplant.

### 3.3. Impact of CMV

Most participants did not experience or recall any symptoms during their episode of CMV viremia (17/25; 85%). Of those who were symptomatic, the majority experienced fatigue, muscle aches, or gastrointestinal symptoms. Participants would often report that they found it difficult to isolate CMV symptoms from general fatigue after transplantation. The impact of CMV diagnosis on mood, work, and social life was minimal. Participants who did express an effect of CMV diagnosis on mood related it to concerns about how the infection may impact the kidney, citing a fear of rejection or early graft loss and having to return to dialysis.

“[Did your CMV Infection affect your mood?] Umm, no, just the just concern about wanting to do everything that I could and to make sure that I was helping the clinic control things really” (CMV4, Male, 67 years)

### 3.4. CMV Management

#### 3.4.1. Effective Treatment With a Tolerable Side Effect Profile

Participants rated the efficacy of their CMV treatment at 9.5/10 (SD = 1.6). 32% of patients who developed CMV viraemia experienced side effects of anti‐CMV therapy (predominantly a course of valganciclovir), most commonly paraesthesia (6/25; 24%), followed by diarrhoea and neutropenia. These side effects were generally deemed acceptable considering the beneficial effects of the CMV treatment.

Patients generally reported that the CMV medication was easy to take and did not represent a significant inconvenience, as they were already taking daily medications. However, two patients reported long waits at the pharmacy as a barrier to receiving treatment.

“It was just another medicine added.” (CMV5, Male, 72 years)

#### 3.4.2. Prophylactic vs Pre‐Emptive Treatment

Only 18% (9/50) of patients were aware of the difference between pre‐emptive and prophylactic CMV management, with 94% (47/50) preferring the pre‐emptive approach due to the heavy medication burden and strict adherence to immunosuppression that they already experience.

“No, it wasn′t explained to me, no. But I mean, I′m quite happy with the way it′s been dealt with in my case.” (CMV24, Male, 58 years)

Moreover, 56% (28/50) of patients felt that more should be done to explain the difference between pre‐emptive and prophylactic treatment.

“Yes. I think it′s a, how do I phrase it, I think it′s a cooperative process between patient and doctor and the patient should not be passive the patient should ensure that they′ve got that they′re taking on the information it′s a shared responsibility” (CMV20, Male, 67 years).

## 4. Discussion

Data capturing the patient experience of CMV in the kidney transplant population is limited, despite its ubiquity and potentially devastating consequences. This study aims to understand awareness of CMV and its management in the kidney transplant community through interviewing kidney transplant recipients who had developed CMV (*n* = 25) and kidney transplant recipients who had not (*n* = 25). We focused on three domains: awareness of CMV and its management, effect of CMV infection on quality of life, and efficacy of current treatment.

This qualitative study underscores the limited awareness surrounding CMV in kidney transplant recipients, with only 40% of participants aware of CMV at the time of interview. This is somewhat a striking statistic as kidney transplant recipients have multiple clinical encounters with nephrologists, surgeons, and clinical nurse specialists leading up to their transplant and opportunities for discussion of the potential risks of transplantation.

Patient difficulty in recalling medical information is a well‐documented phenomenon [17–20]. Several factors within our cohort likely contributed to impaired recall. Notably, 40% of our participants were over the age of 65 at the time of the interview, and advanced age is a well‐established predictor of memory decline [21, 22]. Additionally, anxiety has been shown to inversely affect recall, with higher anxiety levels leading to poorer retention of information [23]. The volume of information provided also plays a role, with a linear relationship between the amount of information given and the proportion remembered [21, 24]. Given the often high‐stress, information‐dense environment patients experience before transplantation, it is unsurprising that recall may be limited.

This awareness gap has significant clinical implications, as it may influence engagement with post‐transplant monitoring, adherence to antiviral therapy, and timely reporting of symptoms. CMV infection and reactivation can be asymptomatic or present with nonspecific features, therefore patient understanding of CMV risk and surveillance may support earlier recognition of potential complications. Improved awareness may also help contextualize the need for repeated blood tests and prolonged prophylaxis, potentially reducing anxiety and improving adherence during the post‐transplant period.

Whilst this is the first study to explore awareness of CMV in the kidney transplant population in the UK, limited awareness of CMV has been reported in a similar study in Lagos, Nigeria, where 40 kidney transplant recipients were evaluated and only 10% were aware of CMV prior to the interviews [25]. Limited awareness on CMV is not limited to the transplantation setting, with poor CMV awareness also reported in the obstetrics literature regarding congenital CMV, with a recent study by Calvert et al. finding that only 36% of pregnant women in the UK were familiar with congenital CMV [26]. Interestingly, there is significant global variation in CMV awareness in the pregnant population, ranging from as low as 11.4% in Ireland to 60% in France [27]. This reinforces that cultural variations, healthcare policy differences, and the presence of targeted educational initiatives play a crucial role in shaping public knowledge.

There was overwhelming agreement that more should be done to improve patient awareness, with 41 out of 50 participants expressing a desire for better education on the risks of CMV infection. The preferred approach was direct, one‐on‐one discussions with healthcare professionals, supplemented by printed materials that could be revisited later. This would likely involve multiple one‐on‐one discussions over the course of years, given that up to 3 years may elapse from a potential transplant recipient being seen in clinic to receiving a transplant [28].

The potential positive impact of multimedia education programs in addressing poor CMV awareness can be seen in the congenital CMV community, where Calvert et al. designed a film‐based digital educational program to increase awareness of CMV among pregnant women in the UK in 2021 [26]. They found that the intervention significantly improved participants’ knowledge about CMV transmission routes, potential health impacts on infants, and preventive behaviours. Furthermore, the intervention group demonstrated meaningful behaviour changes, such as reduced engagement in high‐risk activities like sharing utensils with children. These knowledge gains and behaviour modifications were sustained throughout pregnancy, highlighting the effectiveness of digital educational tools in fostering lasting awareness and risk reduction. The study also emphasized the high acceptability of multimedia interventions, with participants reporting increased confidence in their ability to implement preventive measures and expressing willingness to recommend the program to others [26].

The main strength of our study is the in‐depth interviews performed using a sample of renal transplant recipients who did and not develop CMV viraemia, but there are several limitations. As a single‐centre experience, our findings may not generalize nationally, particularly given the unique “pre‐emptive” approach to CMV management taken at the Royal Free Hospital. Additionally, our participant pool was predominantly White British and all able to speak English fluently, introducing selection bias and limiting our ability to assess CMV awareness in non‐English speaking communities, who are at much higher risk of missing key information about CMV due to the language barrier. Furthermore, Interviews with CMV‐positive participants were, on average, longer than those with non‐CMV participants, which could have limited the potential for thematic depth. However, thematic saturation was reached across both groups, and key themes were evident irrespective of interview duration. Recall bias is also a concern, as many patients reflected that they were inundated with information pre‐ and post‐transplant and therefore may have forgotten details on CMV. As with all interview‐based research, findings may also be influenced by social desirability bias, whereby participants may have shaped their responses in ways they perceived to be acceptable or favourable, in addition to the potential for recall and selection bias.

## 5. Conclusion

Our data represent a real‐world analysis of kidney transplant recipients’ awareness, knowledge and experience of CMV infection. This study highlights the significant gap in awareness and understanding of CMV in this population, with the lack of knowledge about CMV, its symptoms, and potential risks indicating a need for improved education and communication. To address this educational gap, we suggest more one‐on‐one discussions in post‐transplant clinic supplemented with printed reading material and medical infographics, re‐enforcing information that patients have likely forgotten amongst the emotional and physical stress of the peritransplant period.

Further work should explore CMV awareness in more diverse populations and across different healthcare settings to develop universally applicable educational strategies [29].

## Funding

This study was supported by a research grant from Kidney Research UK (“Burden of CMV Infection in Post Kidney Transplant Patients—A Patient Survey,” Ref. no. SP/CMV/2023).

## Disclosure

This work was previously presented as a poster at the American Journal of Transplantation conference proceedings (2025).

## Conflicts of Interest

The authors declare no conflicts of interest.

## Supporting Information

Additional supporting information can be found online in the Supporting Information section.

## Supporting information


**Supporting Information** (1) Interview guide. (2) COREQ guidelines checklist. This file documents the study’s correspondence to the COREQ guidelines. (3) Supporting Table 1: illustrative quotations reflecting participant views across each theme.

## Data Availability

The data that support the findings of this study are available from the corresponding author upon reasonable request.
