# Supplementary material for: A Qualitative Study of Cytomegalovirus Awareness and Experience in Kidney Transplant Recipients
Source: J Transplant. 2026 May 26;2026:1290788. doi: 10.1155/joot/1290788 (PMC13202444; doi:10.1155/joot/1290788)
Supplement: Supplementary file 1 — Supporting Information (1) Interview guide. (2) COREQ guidelines checklist. This file documents the study’s correspondence to the COREQ guidelines. (3) Supporting Table 1: illustrative quotations reflecting participant views across each theme. [file JOOT-2026-1290788-s001.docx]

**Supplementary Materials**

**Supplementary Figure 1. Interview guide.**

**Section 1: Quality of life**

- In general, how would you describe your health?
- How much does your health effect your normal work and social activities?
  - Can you give examples of how your health effects your work?
  - Can you give examples of how your health effects your social life?
- How does your health effect your mood?
  - How regularly do you feel this?
- Do you find yourself worrying about your health?
  - What specifically do you worry about?
  - How regularly?

**Section 2: Quality of life pre and post kidney transplant**

- How has the kidney transplant affected your health? (how you feel physically) (toilet/headaches/pains/sob/swelling/energy)
  - Compared to pre-transplant, how would you describe your health now?
- How has your new kidney affected normal work and social activities?
  - Can you give examples of how your health effects your work?
  - Can you give examples of how your health effects your social life? (examples of things you’ve been able to do post transplant)
- Has your kidney transplant affected your mood?
- How anxious are you about your kidney transplant?
  - What specifically do you worry about?
- Has the 12 months after your kidney transplant been as you imagined?

**Section 3: CMV awareness**

- What do you know about CMV?
  - Where did you learn this? When?
  - What about in the context of kidney transplantation?
- Have you ever received information on the risks of CMV in people with kidney transplants?
  - Before and After transplant
- How worried are you about CMV?
  - How often do you worry? (daily, weekly, monthly, yearly)
  - On a scale of 1-10 how intense is this worry
  - What is the source of the worry?
- Have you ever discussed your personal risk of CMV with a healthcare professional
- Did you know your CMV serostatus (whether you’ve had CMV before) prior to your transplant?
  - How do you feel about that
- What are the symptoms of CMV? (a high temperature. aching muscles. tiredness. a skin rash. feeling sick. a sore throat. swollen glands.)
- If you were to develop a CMV infection, would you recognise the symptoms?
  - If no: does this concern,you?
- Do you think more should be done to educate transplant recipients about the risk of CMV infection?
  - If yes: what would be the most effective way to do so? And when

**Section 4: Impact of CMV (asked only to the CMV cohort)**

- Which symptoms of CMV infection do you suffer from? (reduced activity and mobility, mental fatigue and stress)
  - How often do they effect you? (daily, weekly, monthly, yearly)
  - Generally speaking, how much do they bother you?
  - Which symptom bothers you the most?
  - How do they effect work and social life? Examples?
  - How worried are you about developing further symptoms?
- How has CMV infection affected your mood?
- How is life different compared to before you became aware of your CMV? Examples?
- Do you worry about the effect CMV may have on your kidney?

**Section 5: CMV treatment**

- How effective do you feel current CMV treatments are?
  - How effective was your treatment
  - How effective was it compared to people you know
- How much are you bothered by the side effects of your CMV treatment?
  - Which CMV treatment side effect bothers you the most?
  - Did you feel like you had alternatives
- How much time do you currently spend each day on your treatment? Was that inconvenient
- Did you experience any barriers to receiving your treatment
- Do you feel like you have a good understanding of the difference between pre-emptive and prophylactic CMV management?
  - What was your primary source of this information?
  - Was it ever explained to you by a healthcare professional? Was the explanation adequate or did you leave feeling more confused?
  - Do you think this is a conversation that should be had?
- How do you feel about receiving pre-emptive and not prophylactic CMV management?

**Supplementary Figure 2. *COREQ Guidelines Checklist. This file documents the study’s correspondence to the COREQ guidelines within the manuscript***

**
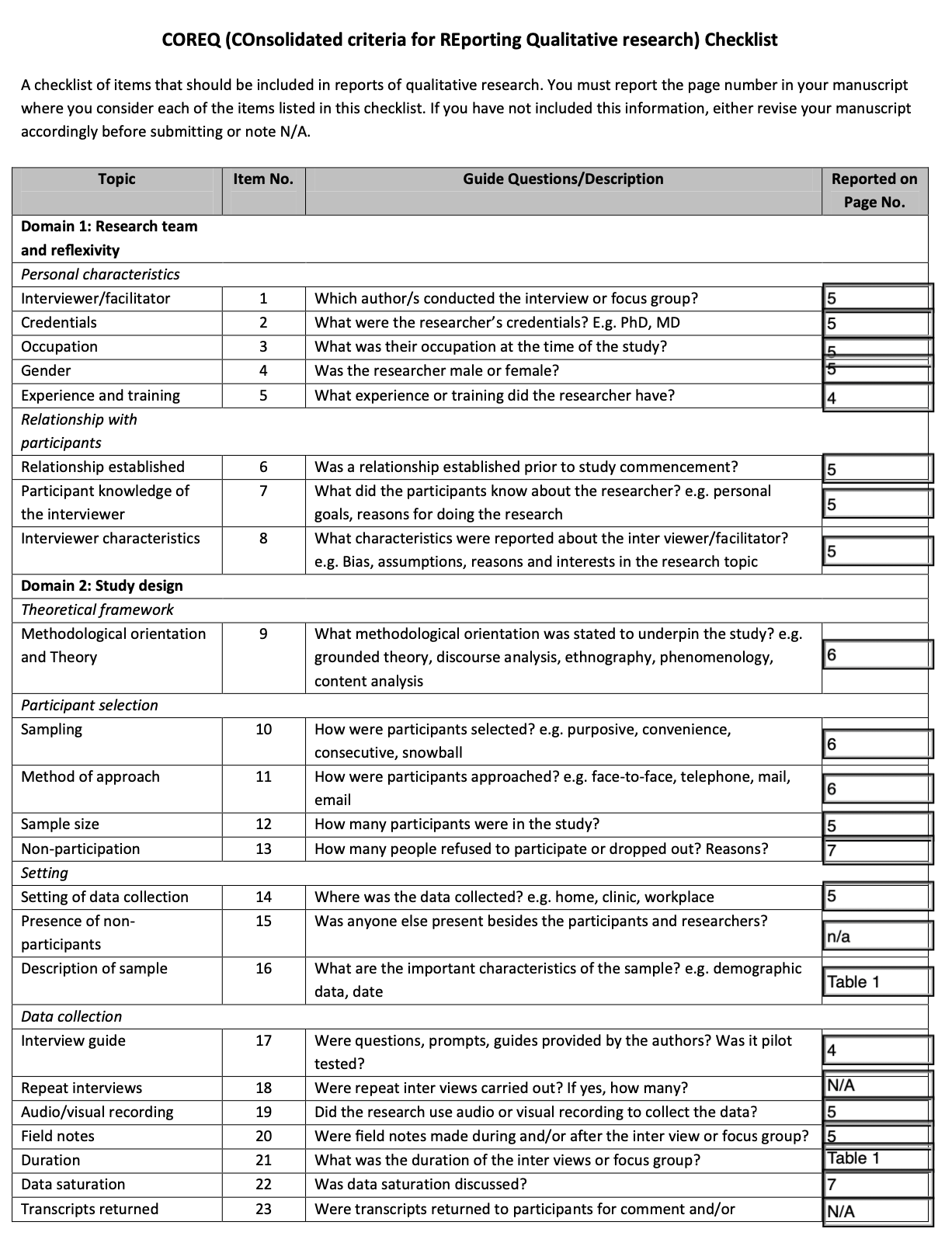
**


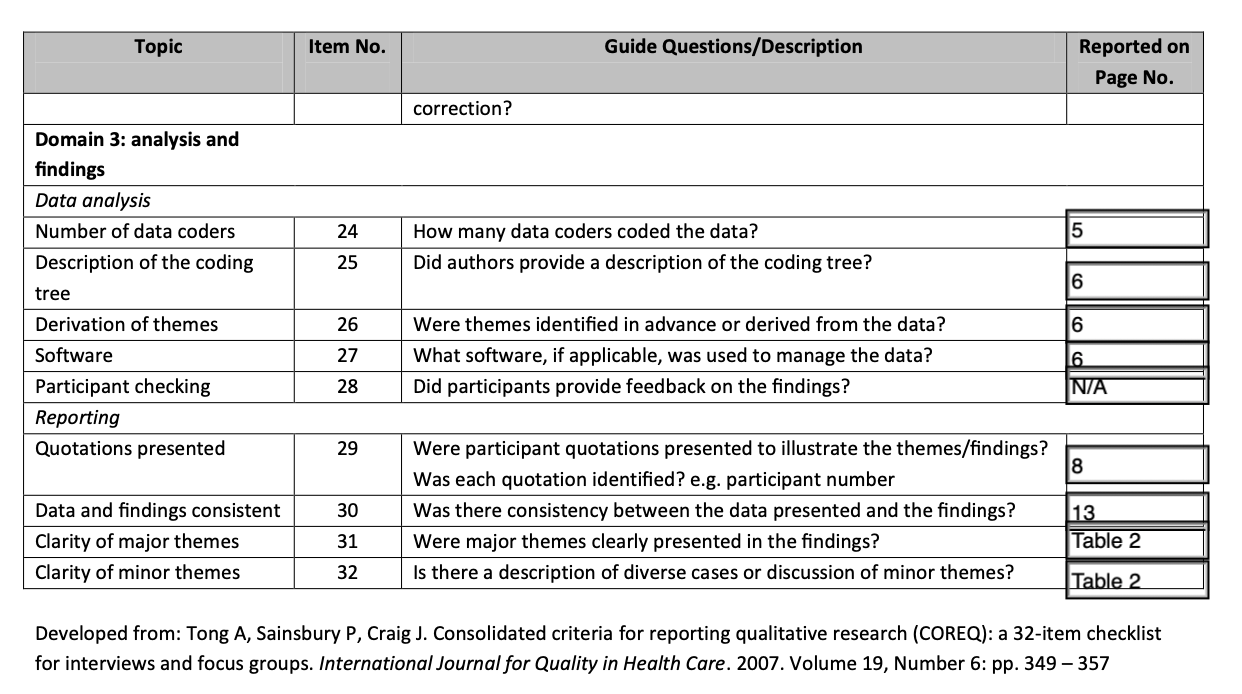


Supplementary Table 1. Illustrative quotations reflecting participant views across each theme

| **Theme** | **Subtheme** | **Quotations** |
| --- | --- | --- |
| Knowledge and awareness of CMV | Limited awareness of CMV | **What do you know about CMV?**  ‘"I knew absolutely nothing about it until we talked last night. " (Non-CMV2)  "When you said CMV I'm like OK, is that something I should know? Because it's not a phrase I know in the same way that I know about immunosuppressants and my post kidney care. "(Non-CMV10)  "I know nothing."(Non-CMV14)  "Well I know that it's a generally harmless virus which a lot of people carry but for people who are immunosuppressed it can be dangerous and that's why it's being taken seriously in my case."(CMV24)  "I only knew about CMV is when they did a blood test and that's when they said that I've got CMV and obviously got it from the transplant. So yeah, I didn't know about it before." (CMV3)  "No I don't [remember CMV] as I had a lot of information as you would imagine over the time, certain things you remember certain things you don't "(Non-CMV10, Male, 57 years) |
|  | Source of knowledge of CMV | **What was the source of your knowledge on CMV?**  "One of the nurses spoke about it. And then when it kind of went a bit sideways, I had to read up a little bit more." (CMV6)  "Uh, my first port of call for things like this is the NHS website, so it was from there.” (Non-CMV6) |
|  | Minimal concern for CMV | **Are you worried about CMV?**  "I'm not really worried because I've got the medications and my CMV levels have been low. I haven't had to take any medication for probably over a year" (CMV15)  "I'm not worried now because it's been negative for quite some time" (CMV3)  "Yeah, I'm not too worried. If I worried about everything and how it might affect me with being immunosuppressed, I'd probably be shut up at home and never go out. So it worries me and I'm aware of it, but I don't think about it too much to be honest." (Non-CMV6) |
|  | Desire for further education regarding CMV | **Should we be doing more to educate people about CMV?**  "Well yeah absolutely, absolutely. I mean it's been what 18 months since I've had mine, no more, 20 months since April 2022. So I certainly I would have liked to have known about it” (Non CMV1)  "I mean yes of course because it's a serious thing” (CMV 5) |
|  | Suggestions for how this education should be delivered | **How should we educate people about CMV?**  "A conversation and a leaflet. Yeah, I think a conversation and a leaflet because, you know, a lot of the NHS websites are hard to navigate. And I can use the computer, I can use my phone and sometimes there's certain things that I find hard to do. So, you know, maybe elderly people, maybe people with disability issues, whatever, it's going to be hard. So a verbal conversation is always going to be the best thing. And then follow up with some information that they can maybe take with them.”(CMV 12)  "So I guess that would be via a conversation and also maybe some follow up material as well. I mean, I got lots of leaflets, I got lots of leaflets during every procedure I went to. Whether I read them all is a different matter, but you know, like, things like, I was given a book on transplants, and I did read that. When you're sitting in a room and someone's telling you for the first time you're getting a transplant, your mind suddenly goes off and thinks about it and not listening to the person talking to you because it's such a major shock. And it's only afterwards that you think about questions that you want to ask and that you go back and you don't always get a chance to go back and talk to the same person because you're seeing a different person every time.” (Non-CMV1)  "Through health care professionals to be honest even a leaflet could help but mainly through health care professionals so when they go to their appointments it should be mentioned." (CMV5) |
| CMV Impact | Minimal physical or affective symptoms | **Did you experience any physical symptoms or changes in mood?**  “I didn't feel anything like I had the virus. I just found out on the test results." (CMV2)  " I had a couple of episodes where you know I just kept on puking feeling sick, I mean I even ended up in the ward and that was probably from the CMV...Yes, I had a little bit of [diarrhoea] every now and then. [the symptoms were] Just [an] inconvenience." (CMV6)  "So you know the diarrhoea, I had a very achy body and I think those were the only two symptoms I had." (CMV21)  " No, I wouldn't say it affected me emotionally, no" (CMV12)  "no I don't think about it." (CMV17) |
|  | Limited concern regarding CMV | **How worried are you about CMV?**  “Not at all. I mean I’ve been monitored every week so I’m not super worried about it. If Dr. X turned around and said that we can’t control it, then yes, then I will be worried, but as long as they can control it then…”(CMV6)  "No, because they're keeping a close eye on me. I go every two weeks and they take a blood test just to monitor if there's anything going on."(CMV19)  "I don’t want a kidney rejection or failing to happen so I'm more worried about that." (CMV5) |
| CMV Treatment | Efficacious therapeutics | **How effective do you think the anti-viral therapies were?**  “ I think they're very effective, I don't know if I would have cleared it alone without the Valganciclovir." (CMV10)  "Yeah, I think it was very effective because, you know, after like a few weeks, it came back down and then I had a recurrence, and I went back on it and it went back down. So it seemed like the medication was working" (CMV15)  "It seems quite random to be honest with me. One medication doesn't work and then change to a different one that works and then that one doesn't work and change back to the other one that works instead and then that doesn't work and then it changes back and back and back and forth." (CMV6) |
|  | Tolerable side effect profile | **How much were you bothered by the side effects of CMV treatment?**  "I don't know if it was the medication but I definitely had a little bit of tingling in the feet." [“: "And did that bother you much?“] "Not really." (CMV14“  "No, because I was so unwell that in fact the treatment could only be a good thing. I was so unwell that I didn't feel any side effects. For me it was all good. " (CMV25)  "I remember getting a slight tingle in my feet and hands. I remember the headaches I was getting and the tummy thing…I could deal with, I'd rather deal with that than yeah, than my copies going up and losing [the graft] " (CMV18, Female, 27 years) |
|  | Prophylactic vs Pre-emptive treatment | **Are you aware of the difference between prophylactic and pre-emptive CMV management?**  "No, it wasn't explained to me, no. But I mean, I'm quite happy with the way it's been dealt with in my case." (CMV24, Male, 58 years) |
